# Exploring the antimicrobial potential of crude peptide extracts from *Allium sativum* and *Allium oschaninii* against antibiotic-resistant bacterial strains

**DOI:** 10.1080/13880209.2024.2395517

**Published:** 2024-08-28

**Authors:** Thitiluck Swangsri, Onrapak Reamtong, Sompob Saralamba, Pakavadee Rakthong, Urusa Thaenkham, Naowarat Saralamba

**Affiliations:** a Department of Molecular Tropical Medicine and Genetics, Faculty of Tropical Medicine, Mahidol University, Bangkok, Thailand; b Mathematical and Economic Modelling (MAEMOD), Mahidol Oxford Tropical Medicine Research Unit, Faculty of Tropical Medicine, Mahidol University, Bangkok, Thailand; c Faculty of Science and Technology, Rajabhat Suratthani University, Surat Thani, Thailand; d Department of Helminthology, Faculty of Tropical Medicine, Mahidol University, Bangkok, Thailand

**Keywords:** Antibacterial activity, natural products, peptide-based antimicrobial

## Abstract

**Context:**

Plant peptides garner attention for their potential antimicrobial properties amid the rising concern over antibiotic-resistant bacteria.

**Objective:**

This study investigates the antibacterial potential of crude peptide extracts from 27 Thai plants collected locally.

**Materials and methods:**

Peptide extracts from 34 plant parts, derived from 27 Thai plants, were tested for their antimicrobial efficacy against four highly resistant bacterial strains: *Streptococcus aureus* MRSA, *Pseudomonas aeruginosa*, *Acinetobacter baumannii*, and *Escherichia coli*. The stability of these peptide extracts was examined at different temperatures, and the synergistic effects of two selected plant peptide extracts were investigated. Additionally, the time-kill kinetics of the individual extracts and their combination were determined against the tested pathogens.

**Results:**

Peptides from *Allium sativum* L. and *Allium oschaninii* O. Fedtsch (Amaryllidaceae) were particularly potent, inhibiting bacterial growth with MICs ranging from 1.43 to 86.50 µg/mL. The consistent MICs and MBCs of these extracts across various extraction time points highlight their reliability. Stability tests reveal that these peptides maintain their antimicrobial activity at −20 °C for over a month, emphasizing their durability for future exploration and potential applications in addressing antibiotic resistance. Time-kill assays elucidate the time and concentration-dependent nature of these antimicrobial effects, underscoring their potent initial activity and sustained efficacy over time.

**Discussion and conclusions:**

This study highlights the antimicrobial potential of *Allium*-derived peptides, endorsing them for combating antibiotic resistance and prompting further investigation into their mechanisms.

## Introduction

The emergence of antibiotic-resistant bacterial strains poses a formidable global health challenge, necessitating innovative approaches to combat these resilient pathogens (Davies and Davies 2010; Chinemerem Nwobodo et al. 2022). As conventional antibiotics face diminishing efficacy, the exploration of alternative antimicrobial agents has gained prominence in recent years (Murugaiyan et al. 2022). One promising avenue of investigation involves the use of bioactive compounds derived from plants, particularly plant-derived peptides, which have demonstrated significant antimicrobial potential (Bahar and Ren 2013; Moretta et al. 2021; Ioannou et al. 2023). Plant antimicrobial peptides (AMPs) provide natural defenses against several kinds of infection and are produced from almost every plant part: leaves, stems, flowers, seeds, and roots (Tam et al. 2015; Wang et al. 2016). Most AMPs have broad-spectrum antimicrobial activity against bacteria, fungi, viruses, and parasites (Marcocci et al. 2018; Zahedifard et al. 2020). They are classified by amino acid sequence similarity, cysteine motifs, peptide disulfide bonds, and secondary structure (Tam et al. 2015; Tang et al. 2018). Plant AMPs are more abundant and diverse than other AMP classes (Dos Santos-Silva et al. 2020), and we speculate that plants harbor many AMPs yet to be discovered and described.

Throughout human history, plants have played a vital role in traditional medicine and cultural practices across diverse societies (Leonti and Casu 2013). The use of plants for medicinal purposes dates back thousands of years, with indigenous and traditional knowledge serving as a rich source of information about the therapeutic properties of various plant species (Najmi et al. 2022). These plants have been integral to the treatment of ailments, the promotion of well-being, and the preservation of cultural heritage. Many studies have investigated the potential health benefits of local plants and vegetables used in Thai traditional medicine. Commonly used plants such as turmeric (*Curcuma longa* L. [Zingiberaceae]), ginger (*Zingiber officinale* Rosc. [Zingiberaceae]), and holy basil (*Ocimum tenuiflorum* L. [Lamiaceae]) have been used to treat a variety of conditions including digestive disorders, respiratory problems, and skin infections (Viyoch et al. 2006; Singh and Chaudhuri 2018; Sharifi-Rad et al. 2020; Akaberi et al. 2021; Ahmed et al. 2022; Hussain et al. 2022; Singh et al. 2022). The citrus fruits (*Citrus aurantifolia* Christm.*, C. limon* (L.) Burm. f.*, C. latifolia* Tanka, and *C. limonia* Osbeck [Rutaceae]) have significant anti-inflammatory and antioxidant effects (Amorim et al. 2016). Local Thais use bitter gourd (*Momordica charantia* L. [Cucurbitaceae]) and cinnamon (*Cinnamomum verum* J. Presl [Lauraceae]), known for their hypoglycemic effects, to effectively manage diabetes (Andrade et al. 2020). A remedy made from the leaves of papaya tree (*Carica papaya* Linn. [Caricaceae]) has antiviral activity against the dengue virus and can potentially treat dengue fever (Dhiman et al. 2022). The study of plant-derived peptides has accelerated because these molecules are less likely to induce resistance than conventional antibiotics (Yu et al. 2018). Their diverse chemical structures and modes of action offer a multifaceted approach to combating antibiotic-resistant bacteria.

Traditional knowledge of medicinal plants often serves as a valuable starting point for modern scientific investigations. The insights gathered from generations of plant usage by local communities provide a foundation for the discovery of novel bioactive compounds and their potential therapeutic applications (Najmi et al. 2022). The selection of the 27 plants for this study originated from a community survey, which involved informative conversations with elderly community members. These discussions provided insights into the traditional uses and the various therapeutic applications of these plants. The selection of twenty-seven plants demonstrates diversity, ranging from herbs like *Zingiber officinale* (ginger) to trees like *Azadirachta indica* A. Juss. (Meliaceae), opening up a vast landscape for exploration. Traditionally used across various cultures, these plants represent a rich reservoir of untapped antimicrobial potential. Researchers have studied some of these plants for their active compounds and antimicrobial activity. *Barringtonia acutangular* (L.) Gaertn. (Lecythidaceae), traditionally used for medicinal purposes in various cultures. Different parts of the plant, including the bark and leaves, have been employed in traditional medicine for treating skin disorders and inflammatory conditions. Some studies have explored the bioactive compounds and found certain secondary metabolites, such as flavonoids and alkaloids (Vien et al. 2017; Van et al. 2020). These compounds are suggested to exhibit antimicrobial activity (Panda et al. 2017). The hydroalcoholic extract of *Sauropus androgynus* L. Merr (Phyllanthaceae), containing a fatty acid ester called ethyl palmitate, showed potent antiviral activity (Sagna et al. 2023). Pentadecane and α-humulene are the major compounds found in *Alpinia galangal* (L.) Wills. (Zingiberaceae) flower and have demonstrated antimicrobial activity (Tang et al. 2018). A derivative pyridone alkaloid, namely 5-methyl-11-(2-oxopyridin-1(2H)-yl) undecaneperoxoic acid, isolated from *Sansevieria trifasciata* Prain (Asparagaceae), demonstrated antibacterial activity against two common bacteria: *Escherichia coli* and *Streptococcus aureus* (Kasmawati et al. 2023). Numerous plant species have been extensively investigated for their antimicrobial properties. While many of these plants have exhibited such activities, some have undergone further characterization to identify their bioactive compounds. Many of these compounds are derived from plant metabolites, with a focus on non-peptidic components.

*Allium* species, commonly known as garlic, onion, and leeks, have been extensively studied for their remarkable antimicrobial activity. The antimicrobial properties of *Allium* spp. are primarily attributed to the presence of sulfur-containing compounds, such as allicin, diallyl sulfide, and diallyl disulfide (Lu et al. 2011; Casella et al. 2013; Bhatwalkar et al. 2021). These bioactive compounds exhibit potent antibacterial, antifungal, and antiviral activities, making *Allium* extracts valuable candidates for natural antimicrobial agents. Furthermore, the characterization of peptides extracted from *Allium spp*. adds a layer of complexity to their antimicrobial potential. A cationic peptide with nine amino acids has been identified from *Allium sativum*, demonstrating antifungal activity (Li et al. 2023). Another peptide, named alicepin, has been isolated from *Allium cepa* L., showing potential as an antifungal peptide (Wang and Ng 2004). The ongoing exploration of the diverse array of bioactive molecules present in these plants highlights their potential. Such research could lead to the development of innovative antimicrobial agents. As the global challenge of antibiotic resistance intensifies, the importance of understanding and harnessing the antimicrobial properties of these natural compounds becomes increasingly crucial.

Given the wealth of traditional knowledge and the persistent global challenge of antibiotic-resistant bacteria, this study seeks to explore the antimicrobial potential of peptide extracts. These extracts are obtained from various parts of traditionally used plants, specifically *Allium sativum* and *Allium oschaninii.* This research integrates traditional plant use with modern scientific methods, aiming to enrich the existing knowledge about plant-derived peptides as potential novel antimicrobial agents. The study also acknowledges the importance of preserving and respecting traditional wisdom while addressing contemporary health challenges.

## Materials and methods

### Collection of plants

Twenty-seven local plants were collected according to their usefulness in traditional Thai medicine (Table 1). Some plants were sourced from private land with the explicit consent of the landowners. Certain plants were acquired from public land with authorization from village heads. Additionally, a portion of the collected plants was procured from the local fresh market. All the plants gathered were native to the Thai region, underscoring the significance of indigenous botanical resources in traditional healing practices. The process complied with Thai government regulations, evidenced by a registration from the Department of Agriculture (Approval No. 0411/2565). All 34 plant parts were cleaned with tap water and then cut into small pieces for peptide extraction.

**Table 1.** Plant species, part used, traditional uses, and sources.

No.	Botanical name	Part used	Traditional used for treatment	Collected date	Collected area		
					District, province	Latitude	Longitude
1	*Barringtonia acutangula*	Leaves	Crumple up and apply to treat smallpox	3 November 2021	Mueang, Surat Thani	9.14	99.33
2	*Sauropus androgynus*	Leaves/stalk	Crumple up and apply to treat mouth ulcers	3 November 2021	Mueang, Surat Thani	9.14	99.33
3	*Limnophila rugose* Merr.	Leaves/stalk	Crush and apply to blister area to heal the wound	26 January 2022	Mueang, Surat Thani	9.14	99.33
4	*Zingiber officinale*	Rhizome	Burn the outer skin rhizome until charcoal and treat herpes wounds	5 February 2022	Bang Kruai, Nonthaburi	13.81	100.45
5	*Alpinia galanga*	Rhizome	Extract rhizome and apply to treat skin diseases	5 February 2022	Bang Kruai, Nonthaburi	13.81	100.45
6	*Boesenbergia rotunda*	Rhizome	Crumple up and apply to the swollen abscess, relieve itching of the scalp	5 February 2022	Bang Kruai, Nonthaburi	13.81	100.45
7	*Beta vulgaris* L.	Rhizome	Boiled with water and drink to treat inflammatory acne	5 February 2022	Bang Kruai, Nonthaburi	13.81	100.45
8	*Helianthus tuberosus*	Rhizome	Eat to support body immunity	5 February 2022	Bang Kruai, Nonthaburi	13.81	100.45
9	*Etlingera elatior*	Flower	Boiled with water and drink to treat rash, crumple up with alcohol and treat itchy rash	5 February 2022	Lomsak, Phetchabun	16.78	101.25
10	*Carica papaya* L.	Fruit/leaves	Crumple up dried leaves and mixed with coconut milk to treat blisters inflammation	21 February 2022	Bang Kruai, Nonthaburi	13.81	100.45
11	*Allium sativum* L.	Clove	Slice thinly and apply to treat acne	21 February 2022	Bang Kruai, Nonthaburi	13.81	100.45
12	*Pandanus amaryllifolius*	Leaves	Crumple up fresh leaves and apply to treat skin diseases	14 February 2022	Bang Kruai, Nonthaburi	13.81	100.45
13	*Allium cepa* L.	Clove	Crumple up and apply to treat insect bite and swollen wound	21 February 2022	Bang Kruai, Nonthaburi	13.81	100.45
14	*Zingiber montanum*	Rhizome	Pounded finely and mixed with salt and camphor to treat purulent area	21 February 2022	Bang Len, Nakhon Pathom	14.02	100.17
15	*Curcuma longa* L.	Rhizome	Pounded finely and apply to treat skin allergy, inflammation, and red rashes	21 February 2022	Bang Kruai, Nonthaburi	13.81	100.45
16	*Aloe vera*	Leaves	Jelly from fresh leaves treat of burns, fresh wounds, and chronic inflammatory lesions	14 February 2022	Bang Kruai, Nonthaburi	13.81	100.45
17	*Sansevieria trifasciata*	Leaves	Grilled leaves over a fire and use to treat bruises.	14 February 2022	Bang Kruai, Nonthaburi	13.81	100.45
18	*Morinda citrifolia*	Leaves	Boiled fresh leaves and drink to treat tuberculosis, relieve aches and reduce fever	14 February 2022	Bang Kruai, Nonthaburi	13.81	100.45
19	*Ledebouria kirkii* Baker.	Leaves	Crumple up fresh leaves and apply to treat skin diseases	14 February 2022	Bang Kruai, Nonthaburi	13.81	100.45
20	*Azadirachta indica*	Leaves	Eat to relieve symptoms of eczema and rashes	21 February 2022	Bang Len, Nakhon Pathom	14.02	100.17
21	*Lasia spinosa (L.) Thwaites*	Leaves/stalk	Boiled and use water for bath, relieve itching from measles, chickenpox.	6 March 2022	Mueang, Surat Thani	9.14	99.33
22	*Pluchea indica* (L.) Less.	Root/leaves/flower	Boiled stems with water, drink to treat blood rashes on the body, red bumps.	3 November 2021	Mueang, Surat Thani	9.14	99.33
23	*Archidendron jiringa* (Jack) I.C. Nielsen	Fruit/leaves	Crumple up and apply on the wound or dermatitis, cure skin diseases.	6 March 2022	Mueang, Surat Thani	9.14	99.33
24	*Diplazium esculentum* (Retz.)	Root/leaves	Crumple up and apply to treat mouth ulcers	6 March 2022	Mueang, Surat Thani	9.14	99.33
25	*Tradescantia spathacea* Swartz.	Leaves	Boiled and drink to relieve sore throat, cough, and ear drop to cure inflammation	14 February 2022	Bang Kruai, Nonthaburi	13.81	100.45
26	*Coccinia grandis*	Leaves	Pounded finely and apply to treat skin allergy	14 February 2022	Bang Kruai, Nonthaburi	13.81	100.45
27	*Allium oschaninii* O. Fedtsch	Clove	Pounded finely and apply to treat acne and dark spots	21 February 2022	Bang Kruai, Nonthaburi	13.81	100.45

### Pathogens tested

The study utilized four drug-resistant bacteria. These included the Gram-positive *S. aureus* MRSA (BAA-1720) and the Gram-negative *P. aeruginosa* (BAA-3197), *A. baumannii* (BAA-1605), and *E. coli* (BAA-2471). All bacterial strains were purchased from American Type Culture Collection (ATCC, Manassas, VA). All bacterial strains were sub-cultured from the original culture. They were stored at −80 °C and maintained on Mueller Hinton Agar (MHA) at 4 °C. Prior to *in vitro* antimicrobial assays, the bacteria were incubated at 37 °C.

### Peptide extraction

Plants were mixed in a 1:6 ratio (*w/v*) with 20 mmol/L phosphate buffer solution (PBS), pH 7.2. The mixture was stirred overnight at 4 °C and then centrifuged at 3000 × *g* for 15 min. Subsequently, the clear aqueous phase was processed through an Amnicon ultra 15 mL centrifugal filter (Merck, USA) to collect peptides with a molecular weight (MW) greater than 5 kDa. To confirm the presence of crude peptides, the extracts were purified using a C18 cartridge (Agilent, CA, USA). This additional purification step aims to selectively isolate peptides from the plant extracts. Each crude extract was then tested for antibacterial activity. Crude extracts showing significant antibacterial activity underwent fractionation and characterization with a Q-TOF mass spectrometer to confirm the presence of antimicrobial peptides.

### In vitro antimicrobial assay

For each peptide extract, we determined the minimum inhibitory concentration (MIC) against the four drug-resistant bacterial strains. Peptide extracts were dissolved in distilled water, and samples from a 2-fold dilution series were mixed with bacterial suspensions at a 1:1 ratio (100 µL total per well). Bacteria were grown overnight in Mueller Hinton Broth (MHB) at 37 °C. Resazurin dye was added to visualize bacterial growth, with the MIC defined as the lowest extract concentration that completely inhibited growth. To determine the minimum bactericidal concentration (MBC), aliquots from the MIC well and two adjacent wells were streaked on MHA plates and incubated for 24 h at 37 °C. The MBC was identified as the lowest extract concentration that killed all bacterial cells.

### Plant peptide stability test

Crude peptide extracts of *A. sativum* and *A. oschaninii* were tested for temperature stability at −20 °C, 4 °C, room temperature (26–30 °C), 37 °C, and 60 °C. Their antimicrobial activity was monitored by measuring MIC and MBC values every 7 days over a month.

### Synergistic effects of plant peptides

Crude peptide extracts from *A. sativum* and *A. oschaninii* were prepared at three different concentration ratios: 1:1X, 0.5:1X, and 1:0.5X, with 1X representing each extract’s MIC. We determined the MICs and MBCs of these mixtures and calculated the fractional inhibitory concentration index (FICI) for each mixture, which measures the MIC ratio of the combination to its individual components. Effects were categorized as synergistic (FICI <0.5), additive (0.5 < FICI <2), or antagonistic (FICI >2) (Elion et al. 1954; Ruden et al. 2009).

### Time-kill assay

We evaluated the time-kill kinetics of *A. sativum*, *A. oschaninii* and their combination at a 1:1X MIC ratio, using 1X, 2X, and 4X MIC concentrations. Each pathogen was prepared to 1.0 McFarland standards, then diluted to an inoculum of 6 × 10^5^ colony forming units (CFU)/mL in a total volume of 5 mL. For controls, we used tubes with only bacteria (positive control) and tubes with only media (negative control). All tubes were incubated at 37 °C. At 0, 1, 2, 4, 6, and 24 h, we sampled 100 µL from each tube, spread it on MHA plates, and incubated these at 37 °C. CFUs were counted after 24 h. The assays, conducted in triplicate, generated data plotted as CFU/mL over time.

## Results

### Identification of plant-derived antimicrobial peptides

We gained knowledge of using local Thai plants to improve health and cure diseases through discussions with local elderly individuals. We collected local plants and documented traditional methods of their use (Table 1). Peptides were successfully extracted from 34 parts of 27 plants. Peptide extracts were tested for antibacterial activity against four drug-resistant bacterial strains. Extracts from *A. sativum* clove and *A. oschaninii* clove inhibited growth of all four pathogens (MICs 1.43–86.50 µg/mL) (Table 2). Extracts from the stalk of *S. androgynus*, the clove of *A. cepa*, and the flower of *P. indica* inhibited growth of *S. aureus* MRSA and *A. baumannii* BAA1605, with MIC values ranging from 2.76 to 176.50 µg/mL. Extract from *L. kirkii* Baker leaves inhibited growth of *P. aeruginosa* BAA3197. Extracts from the leaves of *S. androgynus*, *L. rugose*, *P. amaryllifolius*, *A. vera* leaves, *P. indica*, *T. spathacea*, and *C. grandis* exhibited antimicrobial activity against *S. aureus* MRSA, MICs ranging from 24.88 to 757 µg/mL. The remaining 16 of 34 extracts showed no antimicrobial activity.

**Table 2.** Minimum inhibitory concentration (MIC) and minimum bactericidal concentration (MBC) of crude peptide extracts against the tested bacteria.

No.	Botanical name-part used	Extraction yield (µg/mL)	Tested concentration (µg/ml)	*S. aureus* MRSA (BAA-1720)		*P. aeruginosa* (BAA-3197)		*A. baumannii* (BAA-1605)		*E. coli* (BAA-2471)	
				MIC (µg/ml)	MBC (µg/ml)	MIC (µg/ml)	MBC (µg/ml)	MIC (µg/ml)	MBC (µg/ml)	MIC (µg/ml)	MBC (µg/ml)
1	*Barringtonia acutangula* – leaves	181	90.50-0.35	na	NT	na	NT	na	NT	na	NT
2	*Sauropus androgynus–*leaves	1,514	757-2.96	757	>757	na	NT	na	NT	na	NT
3	*Sauropus androgynus–* stalk	268	134-0.52	67	>134	na	NT	134	134	na	NT
4	*Limnophila rugose* Merr.–leaves	321	160.5-0.63	160.5	>160.50	na	NT	na	NT	na	NT
5	*Limnophila rugose* Merr.–stalk	210	105-0.41	105	>105	na	NT	na	NT	na	NT
6	*Zingiber officinale –* rhizome	30	15-0.06	na	NT	na	NT	na	NT	na	NT
7	*Alpinia galanga–* rhizome	62	31-0.12	na	NT	na	NT	na	NT	na	NT
8	*Boesenbergia rotunda –* rhizome	109	54.5-0.21	na	NT	na	NT	na	NT	na	NT
9	*Beta vulgaris* L. – rhizome	108	54-0.21	na	NT	na	NT	na	NT	na	NT
10	*Helianthus tuberosus –* rhizome	378	189-0.74	na	NT	na	NT	na	NT	na	NT
11	*Etlingera elatior* – flower	173	86.50-0.34	na	NT	na	NT	na	NT	na	NT
12	*Carica papaya* L. – fruit	156	78-0.31	na	NT	na	NT	na	NT	na	NT
13	*Carica papaya* L. –leaves	2,356	1,178-4.60	na	NT	na	NT	na	NT	na	NT
14	*Allium sativum* L. *–*clove	731	365.50-1.43	1.43	5.72	5.72	11.44	1.43	2.86	5.72	11.44
15	*Pandanus amaryllifolius –* leaves	649	324.50-1.63	162.25	>324.50	na	NT	na	NT	na	NT
16	*Allium cepa* L*. –* clove	353	176.50-0.69	2.76	5.52	na	NT	176.50	176.50	na	NT
17	*Zingiber montanum –* rhizome	228	114-0.45	na	NT	na	NT	57	114	na	NT
18	*Curcuma longa* L. – rhizome	1,856	928-3.63	na	NT	928	928	116	464	na	NT
19	*Aloe vera –* leaves	199	99.50-0.39	24.88	>99.50	na	NT	na	NT	na	NT
20	*Sansevieria trifasciata –* leaves	1,042	521-2.04	na	NT	na	NT	na	NT	na	NT
21	*Morinda citrifolia –* leaves	871	435.50-1.70	na	NT	na	NT	435.50	435.50	na	NT
22	*Ledebouria kirkii* Baker. – leaves	571	285.50-1.12	na	NT	142.75	285.50	na	NT	na	NT
23	*Azadirachta indica –* stalk	1,081	540.50-2.11	na	NT	na	NT	na	NT	na	NT
24	*Lasia spinosa (L.) Thwaites –* leaves	296	148-0.58	na	NT	na	NT	na	NT	na	NT
25	*Lasia spinosa (L.) Thwaites –* stalk	635	317.50-1.24	na	NT	na	NT	na	NT	na	NT
26	*Pluchea indica* (L.) Less.– root	235	117.50-0.46	117.50	>117.50	na	NT	na	NT	na	NT
27	*Pluchea indica* (L.) Less. – leaves	199	99.50-0.39	99.5	>99.50	na	NT	na	NT	na	NT
28	*Pluchea indica* (L.) Less. – flower	66	33-0.13	4.13	16.5	na	NT	33	>33	na	NT
29	*Archidendron jiringa* (Jack) I.C. Nielsen–leaves	921	460.50-1.80	na	NT	na	NT	na	NT	na	NT
30	*Archidendron jiringa* (Jack) I.C. Nielsen–fruit	881	440.50-1.72	na	NT	na	NT	na	NT	na	NT
31	*Diplazium esculentum* (Retz.) – root	506	253-0.99	na	NT	na	NT	na	NT	na	NT
32	*Tradescantia spathacea* Swartz. –leaves	360	180-0.70	180	>180	na	NT	na	NT	na	NT
33	*Coccinia grandis –* leaves	378	189-0.74	94.5	>189	na	NT	na	NT	na	NT
34	*Allium oschaninii* O. Fedtsch –clove	692	346-1.35	10.81	43.25	86.50	173	43.25	173	43.25	86.50

* na: not applicable to inhibit growth of the tested bacteria at highest concentration; NT: not tested.

The extracts from different plant sources exhibited varying levels of antimicrobial activity against several bacterial strains. Extracts derived from *A. sativum* and *A. oschaninii* cloves exhibited effectiveness against all four pathogens, with their low MIC values indicating potential for further characterization. A Q-TOF mass spectrometer identified peptide components in crude extracts from both *A. sativum* and *A. oschaninii*, following the determination of active fractions. We analyzed raw data from LC-MS/MS through database comparison, which revealed bioinformatic predictions of antimicrobial peptides in some extracts.

### Plant peptide stability

We assessed the stability of crude peptides extracted from *A. sativum* and *A. oschaninii* over one month at five different temperatures. MICs and MBCs were subsequently retested against *S. aureus* MRSA and *A. baumannii* on days 0, 7, 21, and 30. The antibacterial activity of both peptide extracts remained stable during one month of storage at −20 °C (Table 3). Furthermore, the peptide extract from *A. oschaninii* exhibited consistent antibacterial properties when stored at 4 °C for a month, as indicated by unchanged MIC and MBC values against the tested bacteria. However, extended storage and higher temperatures were observed to adversely affect peptide stability, resulting in higher MIC and MBC values against the tested bacteria (Table 3).

**Table 3.** Minimum inhibitory concentration (MIC) and minimum bactericidal concentration (MBC) of crude peptide from *A. sativum* L. -clove and *A. oschaninii* O. Fedtsch -clove at different temperatures and time periods.

Peptide extract	Temperature (^o^C)	Time (days)	*S. aureus* MRSA (BAA-1720)		*A. baumannii* (BAA-1605)	
			MIC (ug/ml)	MBC (ug/ml)	MIC (ug/ml)	MBC (ug/ml)
*A. sativum* L. -clove	−20	0	2	4	2	4
		7	2	4	2	4
		21	2	4	2	4
		30	2	4	2	4
	4	0	2	4	2	4
		7	4	>16	2	4
		21	4	>16	2	4
		30	4	>16	2	4
	26–30 (Ambient)	0	2	4	2	4
		7	8	>16	4	4
		21	>16	>16	16	16
		30	>16	>16	>16	>16
	37	0	2	4	2	4
		7	16	>16	16	16
		21	>16	>16	>16	>16
		30	>16	>16	>16	>16
	60	0	2	4	2	4
		7	>16	>16	>16	>16
		21	>16	>16	>16	>16
		30	>16	>16	>16	>16
*A. oschaninii* O. Fedtsch -clove	−20	0	12.5	50	50	100
		7	12.5	50	50	100
		21	12.5	50	50	100
		30	12.5	50	50	100
	4	0	12.5	50	50	100
		7	12.5	50	50	100
		21	12.5	50	50	100
		30	12.5	50	50	100
	26–30 (Ambient)	0	12.5	50	50	100
		7	50	100	>100	>100
		21	100	>100	>100	>100
		30	>100	>100	>100	>100
	37	0	12.5	50	50	100
		7	>100	>100	>100	>100
		21	>100	>100	>100	>100
		30	>100	>100	>100	>100
	60	0	12.5	50	50	100
		7	>100	>100	>100	>100
		21	>100	>100	>100	>100
		30	>100	>100	>100	>100

### Antimicrobial impact of peptide combinations from A. sativum and A. oschaninii

We meticulously formulated crude peptide mixtures of *A. sativum* and *A. oschaninii* extracts at varying ratios (1:1X, 0.5:1X, and 1:0.5X the MIC) and subsequently evaluated their MICs and MBCs against four bacterial strains. The majority of these combinations demonstrated heightened potency in comparison to individual peptide samples, as illustrated in Table 4. However, despite the observed improvements, no positive synergistic effects were identified when assessed using the Fractional Inhibitory Concentration Index (FICI). Instead, we detected additive effects at specific ratio: 1:1X MIC of *A. sativum* to *A. oschaninii* against *A. baumannii*; 0.5:1X against *S. aureus* MRSA and *A. baumannii*; and 1:0.5X MIC against *P. aeruginosa*.

**Table 4.** Synergistic antimicrobial effect of combined peptide extracts from *A. sativum* L. and *A. oschaninii* O. Fedtsch against tested bacteria.

Peptide extract	*S. aureus* MRSA (BAA-1720)		*P. aeruginosa* (BAA-3197)		*A. baumannii* (BAA-1605)		*E. coli* (BAA2471)	
	MIC (ug/ml)	MBC (ug/ml)	MIC (ug/ml)	MBC (ug/ml)	MIC (ug/ml)	MBC (ug/ml)	MIC (ug/ml)	MBC (ug/ml)
*A. sativum* L.	2	4	8	16	2	4	8	16
*A. oschaninii* O. Fedtsch	12.5	50	50	50	50	100	50	100
*A. sativum* L.: *A. oschaninii* O. Fedtsch at 1:1X MIC	2.0:12.5	8.0:50.0	8.0:50.0	16.0:100.0	2.0:12.5^b^	4.0:25.0	8.0:50.0	8.0:50.0
*A. sativum* L.: *A. oschaninii* O. Fedtsch at 0.5:1X MIC	1.0:12.5^a^	8.0:100.0	8.0:100.0	8.0:100.0	2.0:25.0^a^	2.0:25.0	8.0:100.0	8.0:100.0
*A. sativum* L.: *A. oschaninii* O. Fedtsch at 1:0.5X MIC	4.0:12.5	16.0:50.0	8.0:25.0^a^	16.0:50.0	4.0:12.5	4.0:12.5	16.0:50.0	16.0:50.0

^a^ FICI = 1.50; ^b^FICI = 1.25, additive effect [FICI is interpreted as synergistic (FICI <0.5), additive (0.5≤ FICI <2), or antagonistic (FICI >2)].

### Time-kill assay

**Figure 1.**
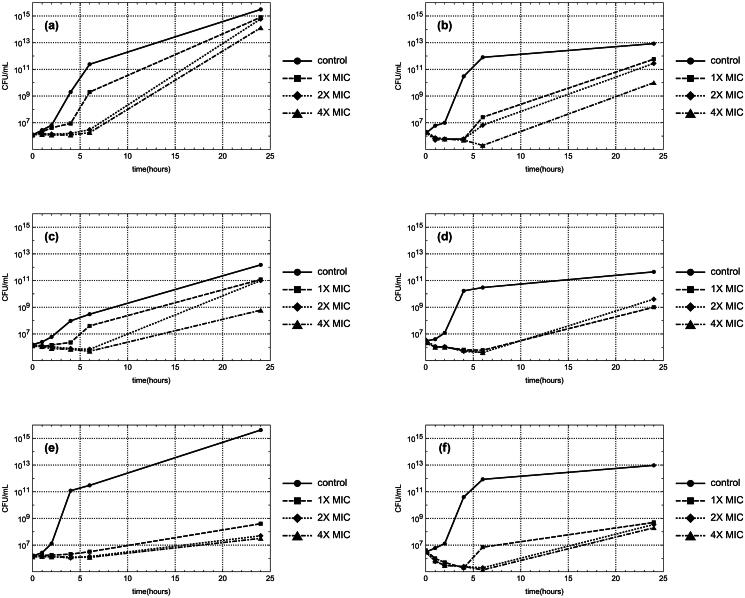
Time-kill curves demonstrating the effects of various treatments: (a) *S. aureus* MRSA treated with *A. sativum* extract. (b) *A. baumannii* treated with *A. sativum* extract. (c) *S. aureus* MRSA treated with *A. oschaninii* extract. (d) *A. baumannii* treated with *A. oschaninii* extract. (e) *S. aureus* MRSA treated with combination of *A. sativum* extract: *A. oschaninii* extract at 0.5:1X MIC. (f) *A. baumannii* treated with combination of *A. sativum* extract: *A. oschaninii* extract at 1:1X MIC. Peptide extract concentrations are indicated by different symbols.

The crude peptides of *A. sativum* and *A. oschaninii* at various concentrations (1X, 2X, and 4X MIC) exhibited inhibitory effects on the growth of *S. aureus* MRSA and *A. baumannii*, particularly within the initial 4–6 h of exposure (Figure 1(a–d)). Notably, *A. oschaninii* crude peptide at 4X MIC displayed complete bactericidal activity against *A. baumannii* at the 24-h mark (Figure 1(d)).

At a 0.5:1X MIC ratio, peptide extracts from *A. sativum* and *A. oschaninii* showed enhanced effectiveness against *S. aureus* MRSA. This combination inhibited bacterial growth for 6 h at concentrations of 1X, 2X, and 4X MIC, though there were some bacterial resurgence after 24 h (Figure 1(e)).

Additionally, a mixture of *A. sativum* and *A. oschaninii* at a 1:1X MIC ratio exhibited varying effects on *A. baumannii*. The 2X and 4X MIC concentrations inhibited growth within 6 h, with limited bacterial regrowth noted after 24 h (Figure 1(f)). Conversely, at 1X MIC, the sample initially inhibited growth within 4 h but permitted some bacterial resurgence at the 6 and 24 h (Figure 1(f)).

## Discussion

In this study, we explored the antimicrobial potential of peptides extracted from various Thai local plants. Traditional knowledge acquired through discussions with local elderly individuals guided our selection of plant sources and traditional curation methods. This guidance was for successful extraction of peptides from multiple plant parts. Notably, extracts from *A. sativum* and *A. oschaninii* cloves exhibited remarkable effectiveness against all four tested pathogens. Both *Allium species* are commonly cultivated worldwide, and their antimicrobial activities were recognized long ago. The characterization of phytochemical compounds revealed the notable involvement of organosulfur compounds in antimicrobial activity (Bhatwalkar et al. 2021). Crude *A. sativum* has shown activity against many drug-resistant bacteria (Magryś et al. 2021). Consider that the crude fresh extract of *A. sativum* exhibited highly effective antibacterial activity against all tested resistant strains, with MIC ranging from 1.43 to 5.72 µg/mL. This is notably lower than previous studies: one reported an MIC of 375 mg/mL for fresh garlic extract against MRSA and *E. coli* (Magryś et al. 2021), and another reported an MIC greater than 7.5 mg/mL for *S. aureus* (Daka 2011). The MIC concentrations in this study, observed in the µg/mL range, are significantly lower than those in previous studies, which were in mg/mL – a thousand-fold difference. The primary reason for this discrepancy may stem from differences in the extraction process. Previous reports indicate that crude extracts were prepared by blending and filtering all components (Daka 2011), potentially yielding peptides in lower concentrations. Additionally, peptide degradation during crude extract production and storage before MIC tests may have contributed to higher MIC values. These variations underscore the importance of standardized extraction methods and careful consideration of sample handling in ensuring accurate and comparable MIC results across studies.

The antimicrobial activity of crude peptides extracted from *A. sativum* and *A. oschaninii* cloves was compared at different extraction time points, and their consistency in activity was demonstrated. Regarding the storage of these crude peptides, the results revealed that the antibacterial activity of both *A. sativum* and *A. oschaninii* cloves remained intact when stored at −20 °C for one month. Furthermore, the peptide extract from *A. oschaninii* exhibited remarkable stability even at 4 °C, with no significant alterations in MIC and MBC values during the same duration. However, it is essential to emphasize that extended storage at higher temperatures adversely affected peptide stability (Akbarian and Chen 2022), leading to increased MIC and MBC values against the tested bacteria.

We also investigated the synergistic potential of peptide combinations from *A. sativum* and *A. oschaninii*. While most combinations exhibited enhanced potency compared to individual peptides, we detected no positive synergistic effects using the Fractional Inhibitory Concentration Index (FICI). Instead, additive effects were observed at specific ratios. This cooperativity suggests that combination agents have the potential to increase efficacy and minimize resistance development in target pathogens. Such findings align with existing notions of combination therapy for antimicrobial treatment (Tängdén 2014; Savoldi et al. 2021). Combinations of antimicrobial peptides (AMPs) have been shown to inhibit cross-resistance and impede the evolution of drug resistance in comparison to individual AMPs (Maron et al. 2022). A fresh extract of *A. sativum*, when combined with gentamicin and ciprofloxacin, inhibit multidrug-resistant bacteria (Magryś et al. 2021). Nevertheless, the peptide composition of *A. sativum* and *A. oschaninii* warrants further research and validation. The detection of additive effects at specific ratios suggests that some combinations of *A. sativum* and *A. oschaninii* peptides may exert complementary or cumulative effects against specific bacterial strains. These additive effects may result from the combined action of peptides targeting different aspects of bacterial physiology or resistance mechanisms. Overall, the findings highlight the importance of systematically evaluating peptide combinations and their potential interactions against bacterial pathogens. While synergistic effects were not observed in this study, the identification of additive effects underscores the potential of combination therapies in combating drug-resistant bacteria. Further investigations into the mechanisms of peptide interactions and their impact on bacterial resistance could yield valuable insights for developing new antimicrobial strategies.

Furthermore, our time-kill assays provided valuable insights into the inhibitory and bactericidal effects of these crude peptides, particularly within the initial 4 to 6 h of exposure. Remarkably, the *A. oschaninii* crude peptide at 4X MIC exhibited complete bactericidal activity against *A. baumannii* within 24 h, demonstrating its potential as an effective antimicrobial agent. The observed antimicrobial activity in this study may be attributed to several potential mechanisms within these plant extracts. Although this study did not explicitly identify the exact constituents responsible for the antimicrobial effects, several plausible mechanisms are worth considering. Firstly, the extracts might contain bioactive compounds like allicin commonly found in *A. sativum*, known to disrupt bacterial cell membranes and interfere with cellular processes, thereby inhibiting microbial growth (Bhatwalkar et al. 2021). Secondly, peptides from these plants may have specific structures that interact with bacterial membranes, disrupting their integrity. Such interaction can increase permeability, cause leakage of cellular contents, and ultimately lead to cell death (Yan et al. 2021). Thirdly, certain components within the plant extracts may act as enzyme inhibitors, disrupting essential bacterial enzymes and metabolic pathways (Dangkulwanich et al. 2019). This interference could impede bacterial growth and survival. Additionally, combining peptides from *A. sativum* and *A. oschaninii* may yield complementary actions that target various stages of bacterial physiology. This synergistic interaction could enhance the overall antimicrobial effect of the formulated crude peptide mixtures. The specific antimicrobial mechanisms can vary depending on the bacterial strain and the unique composition of the plant extracts. The antimicrobial activity observed in this study may be influenced by the synergy or additive effects of multiple bioactive compounds working in concert. Further investigations into the specific constituents of these extracts and their individual and combined effects will deepen our understanding of the mechanisms underlying the observed antimicrobial activity.

Overall, our study has unveiled the promising antimicrobial properties of peptides derived from Thai local plants, emphasizing their potential application in combatting drug-resistant bacterial strains. These findings open avenues for further research to refine peptide combinations and optimize their effectiveness for future therapeutic and pharmaceutical purposes, contributing to the ongoing battle against antibiotic-resistant bacteria. Further research, including *in vivo* studies and toxicity evaluation, is essential to advance these plant-derived peptides as effective antimicrobial agents.

## Conclusions

This study investigated the antimicrobial properties of peptide extracts derived from various Thai local plants. *In vitro*, the extracts, particularly those from *A. sativum* and *A. oschaninii,* showed significant activity by inhibiting the growth of all tested bacteria. The stability assessment of crude peptides demonstrated that they retained their antibacterial activity during one month of storage at −20 °C. The combined use of extracts from *A. sativum* and *A. oschaninii* demonstrated enhanced effectiveness against certain bacteria. These results provide valuable insights, underscoring the potential of these peptides to combat drug-resistant bacteria and highlighting the importance of optimizing peptide combinations for better antimicrobial effectiveness.

## Data Availability

The data used to support the findings of this study are available from the corresponding author upon request.
